# Supplementary material for: Predicting visual function by interpreting a neuronal wiring diagram
Source: Nature. 2024 Oct 2;634(8032):113–23. doi: 10.1038/s41586-024-07953-5 (PMC11446822; doi:10.1038/s41586-024-07953-5)
Supplement: Supplementary file 6 — CRF and ERF predictions for individual TmY4 and TmY9 cells. Analogous to Supplementary Data 3, but for TmY target types. Shown are the top four monosynaptic pathways, the strongest pathway passing through each of the top ten intermediary types (ranking from Extended Data Fig. 7), and the trisynaptic pathway Tm1–TmY–Dm3–TmY (see the section entitled Prediction of spatial normalization). [file 41586_2024_7953_MOESM6_ESM.zip › DataS4/TmY9qΓèÑ/720575940615730754.pdf]

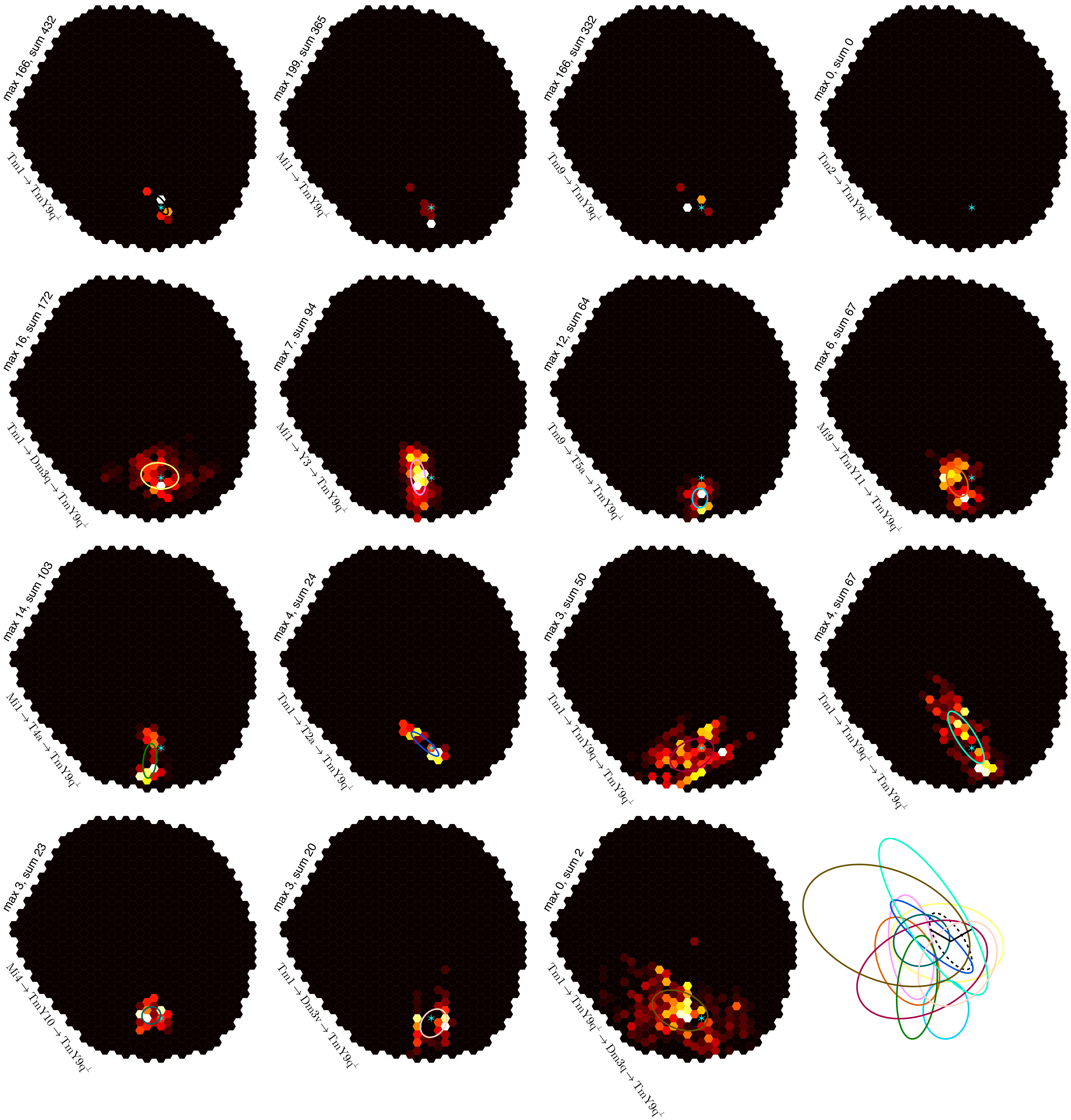

max 166, sum 432
Tm1 → TmY9q⊥
max 199, sum 365
Mi1 → TmY9q⊥
max 166, sum 332
Tm9 → TmY9q⊥
max 0, sum 0
Tm2 → TmY9q⊥
max 16, sum 172
Tm1 → Dm3q → TmY9q⊥
max 7, sum 94
Mi1 → Y3 → TmY9q⊥
max 12, sum 64
Tm9 → T5a → TmY9q⊥
max 6, sum 67
Mi9 → TmY11 → TmY9q⊥
max 14, sum 103
Mi1 → T4a → TmY9q⊥
max 4, sum 24
Tm1 → T2a → TmY9q⊥
max 3, sum 50
Tm1 → TmY9q → TmY9q⊥
max 4, sum 67
Tm1 → TmY9q⊥ → TmY9q⊥
max 3, sum 23
Mi4 → TmY10 → TmY9q⊥
max 3, sum 20
Tm1 → Dm3v → TmY9q⊥
max 0, sum 2
Tm1 → TmY9q⊥ → Dm3q → TmY9q⊥
